# Fibrin degradation products and survival in patients with chronic obstructive pulmonary disease: a protocolized prospective observational study

**DOI:** 10.1186/s12931-023-02472-9

**Published:** 2023-06-27

**Authors:** Peter Kamstrup, Pradeesh Sivapalan, Christian Rønn, Ema Rastoder, Daniel Modin, Anna Kjaer Kristensen, Elisabeth Bendstrup, Rikke Sørensen, Tor Biering-Sørensen, Charlotte Suppli Ulrik, Jørgen Vestbo, Jens-Ulrik Jensen

**Affiliations:** 1 Section of Respiratory Medicine, Copenhagen University Hospital Herlev and Gentofte, Hellerup, 2900 Denmark; 2 Department of Clinical Medicine, University of Copenhagen, Copenhagen, 2200 Denmark; 3 Department of Respiratory Medicine, Copenhagen University Hospital-Hvidovre, Hvidovre, 2650 Denmark; 4 The North West Lung Centre, Wythenshawe Hospital, Manchester University NHS Foundation Trust, Manchester, M13 9PL UK; 5 Division of Infection, Immunity and Respiratory Medicine, School of Biological Sciences, The University of Manchester, Manchester Academic Health Science Centre, Manchester, M13 9PL UK; 6 Department of Cardiology, Copenhagen University Hospital Rigshospitalet, Copenhagen, 2100 Denmark; 7 Department of Cardiology, Copenhagen University Hospital Herlev and Gentofte, Hellerup, 2900 Denmark; 8 Department Respiratory Disease and Allergy, Aarhus University Hospital, Aarhus, 8000 Denmark; 9 Department of Clinical Medicine, Aarhus University, Aarhus, 8200 Denmark

**Keywords:** Cohort, D-dimer, COPD, Biomarker, All-cause mortality

## Abstract

**Background:**

Patients with chronic obstructive pulmonary disease (COPD) have a high incidence of cardiovascular disease including thromboembolisms. Fibrin degradation products, like D-dimer, have been associated with death from all causes in healthy individuals and COPD patients. We aimed to determine the (i) association between D-dimer levels and all-cause mortality and time being alive and out of a hospital, (ii) possible modifying effect of anticoagulant treatment,, and (iii) distribution of D-dimer in patients with moderate to severe COPD.

**Methods:**

Results of routinely measured stable phase D-dimer samples from COPD-outpatients at Copenhagen University Hospital – Herlev and Gentofte, COPD-outpatient clinic were collected using the Danish registries. These were used to examine whether COPD-patients with a D-dimer level in the upper quartile, had a higher risk of death from all causes within 365 days.

**Results:**

In the unadjusted Cox proportional hazards regression we found an association between high D-dimer and all-cause mortality: Hazard ratio (HR): 2.3 (95% Confidence Interval (CI) 1.1–4.7). In the fully adjusted regression, the HR was 1.8 (CI 0.8–3.9). We did not find any interaction between D-dimer and anticoagulant or antiplatelet therapy. For the secondary outcome, proportion of days alive and out of hospital in 365 days (pDAOH), the unadjusted multiple linear regression had an association between high D-dimer level and pDAOH: -2.7% points (pp) (CI -3.9 pp - -1.5 pp), which was attenuated to -1,7pp (-2.9pp – -0.4pp) in the fully adjusted regression.

**Conclusions:**

In patients with moderate to severe COPD, patients with a high level of D-dimer were more likely to die; however, the signal was not strong in the adjusted analyses and our results do not support unselected risk stratification with D-dimer in COPD-outpatients.

**Supplementary Information:**

The online version contains supplementary material available at 10.1186/s12931-023-02472-9.

## Introduction

Chronic obstructive pulmonary disease (COPD) is a common disease, affecting millions of people all over the world [1]. Patients with COPD have an increased risk of cardiovascular diseases (CVD) and venous thromboembolism (VTE), and these events are common during admissions with acute exacerbations of COPD [2–4]. In addition, COPD patients with VTE have longer admission times and more frequently require mechanical ventilation than COPD patients without VTE [5]. Furthermore, COPD patients with VTE have a higher mortality than COPD patients without VTE [6, 7].

The higher rates of CVD and VTE in patients with COPD has been hypothesized to be a consequence of increased activation of the coagulative pathways, which is likely to be preceded by either systemic inflammation, hypoxia, platelet activation and/or oxidative stress [8]. An increased activation of both coagulative pathways is illustrated by previous findings of elevated factor II, V, VII, VIII and IX, as well as elevated fibrinogen in patients with COPD compared with controls [9, 10]. One of the final products resulting from activation of either of the coagulative pathways is D-dimer, which is a fragment from the degradation of cross-linked fibrin. D-dimer is primarily used because of its high sensitivity for venous thromboembolisms in low-risk populations [11].

Previous studies examining D-dimer levels in stable patients with COPD are conflicting, reporting both higher and comparable levels between COPD patients and controls [12–14]. D-dimer levels have, however, repeatedly been found to be higher during acute exacerbation compared to stable COPD [13, 15–17].

In healthy middle-aged adults, D-dimer in the upper quartile predicts all-cause mortality [18]. Furthermore, in a population without known cardiovascular disease, D-dimer is associated with both all-cause mortality and cancer mortality [19].

In patients admitted with acute exacerbation of COPD, two studies showed that D-dimer predicted both in-hospital and one-year mortality [20, 21], whereas another study failed to show any association between D-dimer and 90-days mortality [22].

For stable COPD patients, an association has been established between D-dimer and mortality, especially cardiovascular and pulmonary mortality [13]. However, D-dimer did not predict time to first exacerbation [13].

In the present study, we aim i) to describe the distribution of D-dimer among outpatients with moderate to severe COPD and whether this is affected by smoking status or anticoagulant or antiplatelet therapy, ii) and whether these possible associations are modified by anticoagulant or antithrombotic treatment, and iii) to investigate whether D-dimer in the highest quartile is associated with all-cause mortality and days alive and out of hospital.

## Methods

### Study design

After we collected stable phase D-dimer values from COPD-outpatients at the COPD outpatient clinic at Copenhagen University Hospital – Herlev and Gentofte as part of the routine examination, we used the Danish registers to obtain the values. Since D-dimer testing were performed in all out-patient COPD visits at this clinic, this led to a cohort of unselected COPD patients from Copenhagen University Hospital – Herlev and Gentofte. More specifically, the study population was defined as all COPD patients with a D-dimer sample collected between the 1st of June 2020 and 21st of April 2023 by the outpatient clinic, Section of Respiratory Medicine, Department of Medicine, Herlev and Gentofte Hospital. The observation period starts on the date of the first out-patient D-dimer sample taken on each patient (after 1st of June 2020) and continues until either *i)* death, *ii)* emigration from Denmark, *iii)* 365 days from the sampling date, or *iv)* 21st of April 2023.

Inclusion criteria were a registered diagnosis of COPD (ICD-10: DJ44) and an outpatient D-dimer sample collected during stable phase at the outpatient clinic. Stable phase was defined as no admission, no antibiotics (except for azithromycin) or prednisolone prescriptions redeemed within 7 days of the D-dimer sample date, to rule out patients who had the D-dimer taken because of acute events.

Exclusion criteria were: 1) Hospital contact for the primary diagnosis of any malignant disease within 2 years prior to the sample date (except non-melanoma malignancies of the skin), 2) known abdominal aortic aneurism or aortic dissection, 3) known VTE within 3 months prior to the sample date, 4) age < 50 years old (to rule out pregnancy), 5) moderate to severe liver disease (See Additional File 1 for definition), 6) surgery within 14 days prior to the sample date, and 7) significant bleeding episode (i.e. requires hospital contact) within 3 months prior to the study.

### Data sources

Data were obtained from the Danish National Patient Registry [23], which contains information on all admissions to Danish Hospitals and out-patient specialist clinic visits, with codes of diagnosis. This was linked with *1*) the National Laboratory Database [24], which contains information on laboratory values from four of five Regions of Denmark, *2)* the National Prescription Registry [25], which contains information on all prescriptions collected in Denmark, and *3)* the Danish Central Person Registry [26], which includes information on citizens of Denmark, including sex, and vital status.

Baseline diagnoses were included if they ever appeared prior to inclusion. Collected prescriptions used for baseline were included if they were collected within 90 days prior to inclusion, except for inhaled medications which were included if they were collected within 182 days prior to inclusion.

International Classification of Disease-10 (ICD-10) codes and Anatomical Therapeutic Chemical (ATC) classification codes used for baseline data are supplied in Additional File 1.

### Power

The power calculation was based on the following assumptions: Mortality of 5% and 15% (corresponding to a hazard ratio (HR) of 3) comparing D-dimer levels in the lower ¾ and upper ¼ quartiles, respectively. Sample allocation ratio 3:1. α: 0.05 and β: 0.80. We estimated the needed observation time to be 336 (252 + 84) person-years follow-up.

### Measurements

D-dimer sampling was performed routinely at the site in the study period. The D-dimer assay used at Herlev and Gentofte Hospital during the study period was the Siemens INNOVANCE D-dimer assay. D-dimer was measured as Fibrin Equivalent Units (FEU).

### Outcomes

Primary outcome:

All-cause mortality.

Secondary outcomes:

1) Days alive and out of hospital in a year (as percentage of follow-up (pDAOH), which was done to utilize patients who were sampled after the 21st of April 2022 and thereby could not contribute 365 days possible follow-up). In-hospital was defined as any contact with a hospital lasting over 12 hours. Outcomes for days alive and out of hospital for participants with 365 days follow-up were reported in additional material.

2) The distribution of D-dimer.

3) D-dimer levels across smoking status.

4) D-dimer levels across users of anticoagulant and antiplatelet therapy.

Both the primary outcome and the first secondary outcome were examined comparing the highest quartile of D-dimer levels to the three lower quartiles, following results previously reported in healthy adults by Di Castelnuovo et al [18].

Post-hoc, mortality across the four D-dimer quartiles was illustrated a Cochran-Armitage Trend test computed.

### Statistical analysis

Descriptive statistics were performed on baseline data to describe the D-dimer levels of patients with severe COPD. Furthermore, the distribution of D-dimer between the subgroups of 1) patients with- and without anticoagulant or P2Y12 inhibitor treatment and 2) smoking status (previous smokers, never smokers and current smokers) was examined.

The threshold for the longitudinal analyses was determined by the descriptive statistics, as the aim was to compare the highest quartile to the rest.

All-cause mortality was examined by log-rank test, and three Cox proportional hazards regressions: An unadjusted regression, a regression adjusted for age and sex, and finally a regression adjusted for age, sex, systemic corticosteroid consumption, inhaled corticosteroids (ICS) consumption and C-reactive protein (CRP). The proportional hazards assumption was tested by adding an interaction with time. Linearity was tested by including each continuous variable squared.

Proportion of days alive and out of hospital was analyzed with a Wilcoxon rank-sum test, on account of the distribution of data followed by linear regression analyses adjusted for the same variables as previously described for the Cox proportional hazards regressions.

The effect of anticoagulant treatment and P2Y12-inhibitor treatment on the association between D-dimer and all-cause mortality was examined, by examining interactions.

For the third and fourth secondary aims, comparisons of D-dimer levels across 1) smoking status (current smokers, former smokers and never smokers) and 2) use of anticoagulant and antiplatelet therapy were performed with Kruskal-Wallis and Wilcoxon Rank-Sum test, respectively. Both were illustrated in box-and-whiskers plots.

Data management, descriptive, comparative statistics and comparative graphs were performed with Statistical Analysis Software 9.4 (SAS Institute, Cary, NC, USA). Multiple imputations and combination of results were conducted in R 4.2.2 (R Foundation for Statistical Computing, Vienna, Austria) with the SMCFCS 1.6.1, and MITOOLS 2.4, packages, respectively. Crude Cox’ proportional hazards regression model was conducted with the SURVIVAL 3.3-1 package. Nelson-Aalen estimator was illustrated using the GGPLOT2 3.4.0-package.

Conversion of absolute forced expiratory volume in one second (FEV_1_) values from liters to percent of expected was done using the Global Lung Institute SAS macro.

A study-protocol was published online (http://coptrin.dk/wp-content/uploads/2022/10/Protokol-ddimer-sign.pdf) prior to conduction of the study.

Reporting of the current study adheres to the STROBE (STrengthening the Reporting of OBservational studies in Epidemiology) guidelines [27].

## Results

**Fig. 1:**
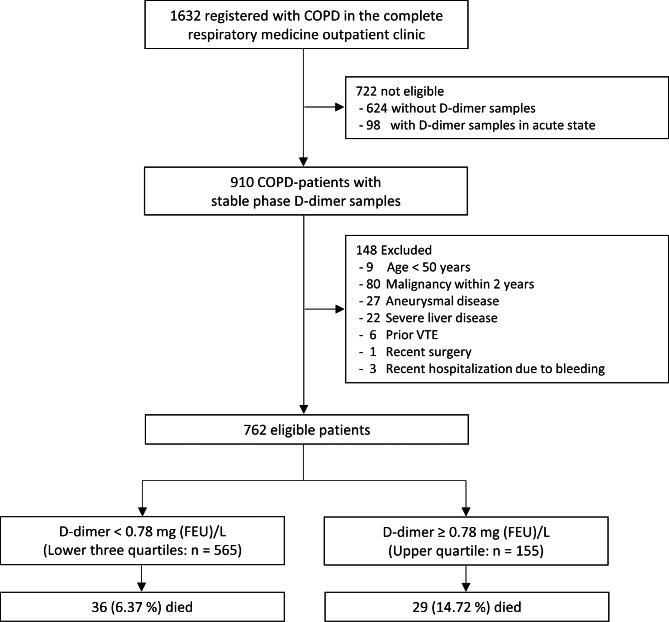
Study flowchart. (Study flowchart. VTE: Venous thromboembolism, FEU: Fibrin equivalent units, n: Number)

In total, 762 patients met the eligibility criteria (Fig. 1). The median and interquartile range (IQR) for D-dimer were: 0.45 mg (0.30–0.78) mg (FEU)/L. Dichotomization at the upper quartile led to 197 patients in the ‘High D-dimer level’ group and 565 in the ‘Low D-dimer level’ group. Two patients migrated from Denmark during the study period and was censored at the time of migration. Baseline data is presented in Table 1.

**Table 1:** Baseline data

	Total (n = 762)	Low D-dimer level (n = 565)	High D-dimer level (n = 197)	Missing data, n (%)	
				Low level	High level
Sex, (female, n (%))	422 (55.4)	310 (54.9)	112 (56.9)	0	0
Age, mean (SD)	71.8 (9.3)	70.5 (9.3)	75.4 (8.1)	0	0
FEV_1_% of predicted, median (IQR)	40.5 (30.1–55.3)	40.9 (29.3–55.5)	39.6 (32.8–55.2)	62 (11.0)	27 (13.7)
BMI, median (IQR)	25.1 (21.5 − 29.6)	25.3 (21.5–29.7)	24.4 (21.3–29.4)	67 (11.9)	29 (14.7)
Smoking status				65 (11.5)	28 (14.2)
- Current smokers	203 (26.6)	155 (27.4)	48 (24.4)		
- Former smokers	445 (58.4)	332 (58.8)	113 (57.4)		
- Never smokers	21 (2.8)	13 (2.3)	8 (4.1)		
D-dimer mg (FEU)/L, median (IQR)	0.5 (0.3–0.8)	0.4 (0.3–0.5)	1.2 (1.0–1.8)	0	0
CRP mg/L, median (IQR)	4 (4.0–9.0)	4.0 (3.0–5.0)	7.0 (4.0–19.5)	4 (0.7)	1 (0.5)
Myocardial infarction, n (%)	86 (11.3)	62 (11.0)	24 (12.2)	0	0
Congestive heart failure, n (%)	122 (16.0)	88 (15.6)	34 (17.3)	0	0
Atrial fibrillation or flutter, n (%)	130 (17.1)	104 (18.4)	26 (13.2)	0	0
Cerebrovascular disease, n (%)	99 (13.0)	72 (12.7)	27 (13.7)	0	0
Dementia, n (%)	8 (1.0)	≤ 5 (≤ 0.9)	≤ 5 (≤ 2.5)	0	0
Peptic ulcer disease, n (%)	44 (5.8)	32 (5.7)	12 (6.1)	0	0
Rheumatic disease, n (%)	63 (8.3)	36 (6.4)	27 (13.7)	0	0
Diabetes mellitus (any type), n (%)	91 (11.9)	55 (9.7)	36 (18.3)	0	0
Moderate to severe renal disease, n (%)	27 (3.5)	11 (1.9)	16 (8.1)	0	0
Prior malignancy, n (%)	80 (10.5)	56 (9.9)	24 (12.2)	0	0
Anticoagulant treatment, n (%)	128 (16.8)	105 (18.6)	23 (11.7)	0	0
Antiplatelet treatment, n (%)	139 (18.2)	89 (15.8)	50 (25.4)	0	0
- Acetylsalicylic acid, n (%)	96 (12.6)	61 (10.8)	35 (17.8)	0	0
- P2Y12-inhibitors, n (%)	51 (6.7)	34 (6.0)	17 (8.6)	0	0
Prednisolone ≥ 25 mg/dose, n (%)	131 (17.2)	98 (18.3)	33 (16.8)	0	0
Prednisolone < 25 mg/dose, n (%)	26 (3.4)	14 (2.5)	12 (6.1)	0	0
Inhaled corticosteroid treatment, n (%)	411 (53.9)	304 (53.8)	107 (54.3)	0	0
Inhaled long-acting muscarinic antagonist, n (%)	342 (44.9)	256 (45.3)	86 (43.7)	0	0
Inhaled long-acting β2-agonist, n (%)	378 (49.6)	279 (49.4)	99 (50.3)	0	0

Baseline data. n: Number, SD: Standard deviation, FEV1: Forced Expiratory Volume in one second, IQR: Interquartile range, FEU: Fibrin equivalent units.

### Primary outcome

The 762 patients contributed 625 person-years of follow-up. In total 65 (8.5%) died, with 29 (14.7%) and 36 (6.4%) in the high and low D-dimer level groups, respectively.

**Fig. 2:**
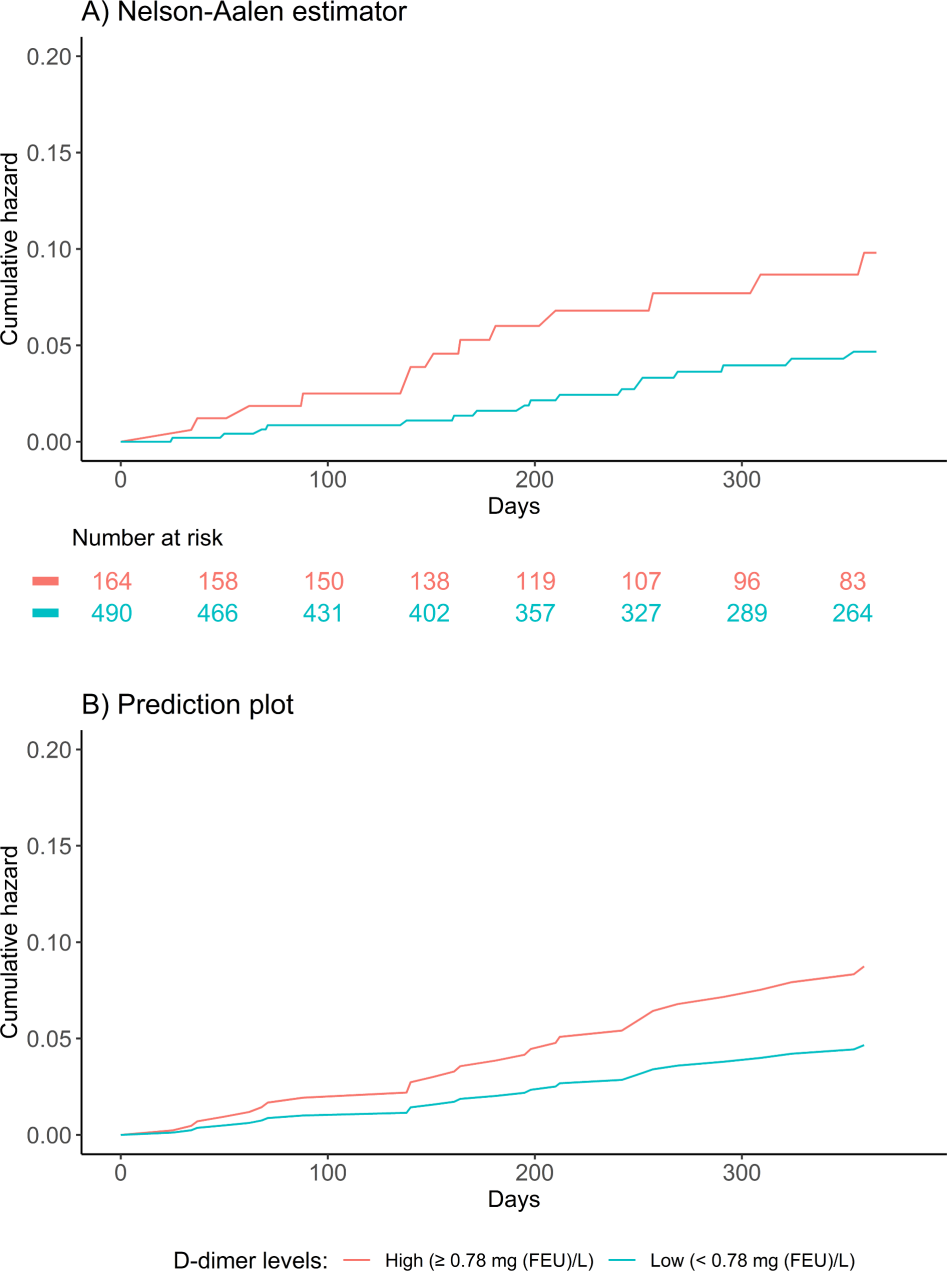
Cumulated incidence for all-cause mortality in high and low levels of D-dimer. (Aalen-Johansen estimator for 365-day cumulated incidence in high (upper quartile) and low (lower three quartiles) D-dimer levels. P-value from log-rank test. FEU: Fibrin equivalent units)

Unadjusted Cox proportional hazards regression showed a HR 2.27 (95% Confidence Interval (CI) 1.10–4.68, p = 0.026) for all-cause mortality. When adjusting for age and sex the HR was 1.99 (0.96–4.18, p = 0.066). For the fully adjusted model (adjusted for age, sex, CRP, oral corticosteroid use, and ICS use) the HR was 1.81 (0.84–3.90, p = 0.13). Figure 2 illustrates Nelson-Aalen estimator (A) and prediction plot of the fully adjusted model (B).

### Model validation

Testing for proportional hazards as well as linearity did not reveal any modelling issues. When testing the interaction between anticoagulant treatment and D-dimer level, the HR comparing high and low D- levels was amplified to 2.15 (0.92–5.02, p = 0.076). See Additional File 2 for the full information on the Cox proportional hazards models.

### Secondary outcomes

#### Days alive and out of hospital

In the total population, the median pDAOH was 100% (interquartile range (IQR) 98.3% − 100.0%). Median (IQR) pDAOH were 99.5% (96.2%-100.0%) in the high D-dimer level group, and 100.0% (98.9%-100.0%) in the low D-dimer level group with a p-value comparing the two groups of < .0001. The unadjusted linear regression estimate was a difference of -2.72 percentage points (pp) (95% CI -3.92 pp- -1.52 pp, p < 0.001) between the high- and low-level groups. Adding age and sex to the regression changed the estimate to -2.50 pp (-3.73 pp- -1.27 pp, p = < .001). When fully adjusted (age, sex, CRP, oral corticosteroid use, and ICS use), the regression the estimate was − 1.65 pp (-2.90 pp - -0.41 pp, p = 0.009). Neither addition of interaction between D-dimer and anticoagulant treatment nor interaction between D-dimer and P2Y12-inhibitor treatment changed the results. See Additional File 3 for the full information on the linear regression analyses. See Additional File 4 for the full information on the linear regression analyses on the individuals with 365 days possible follow-up.

D-dimer level compared to smoking status and use of antiplatelet and anticoagulant treatment

**Fig. 3:**
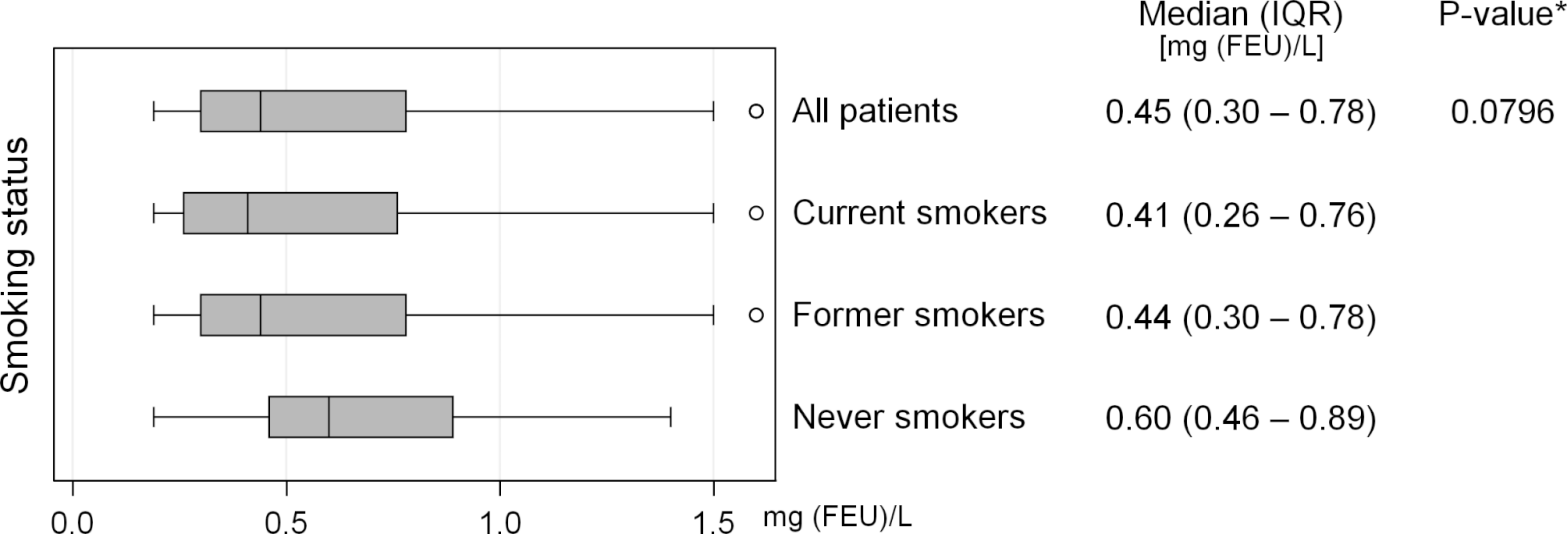
D-dimer levels compared to smoking status. (Box and whiskers plot showing D-dimer levels across smoking status. IQR: Interquartile range. FEU: Fibrin equivalent units. *Kruskal-Wallis test)

**Fig. 4:**
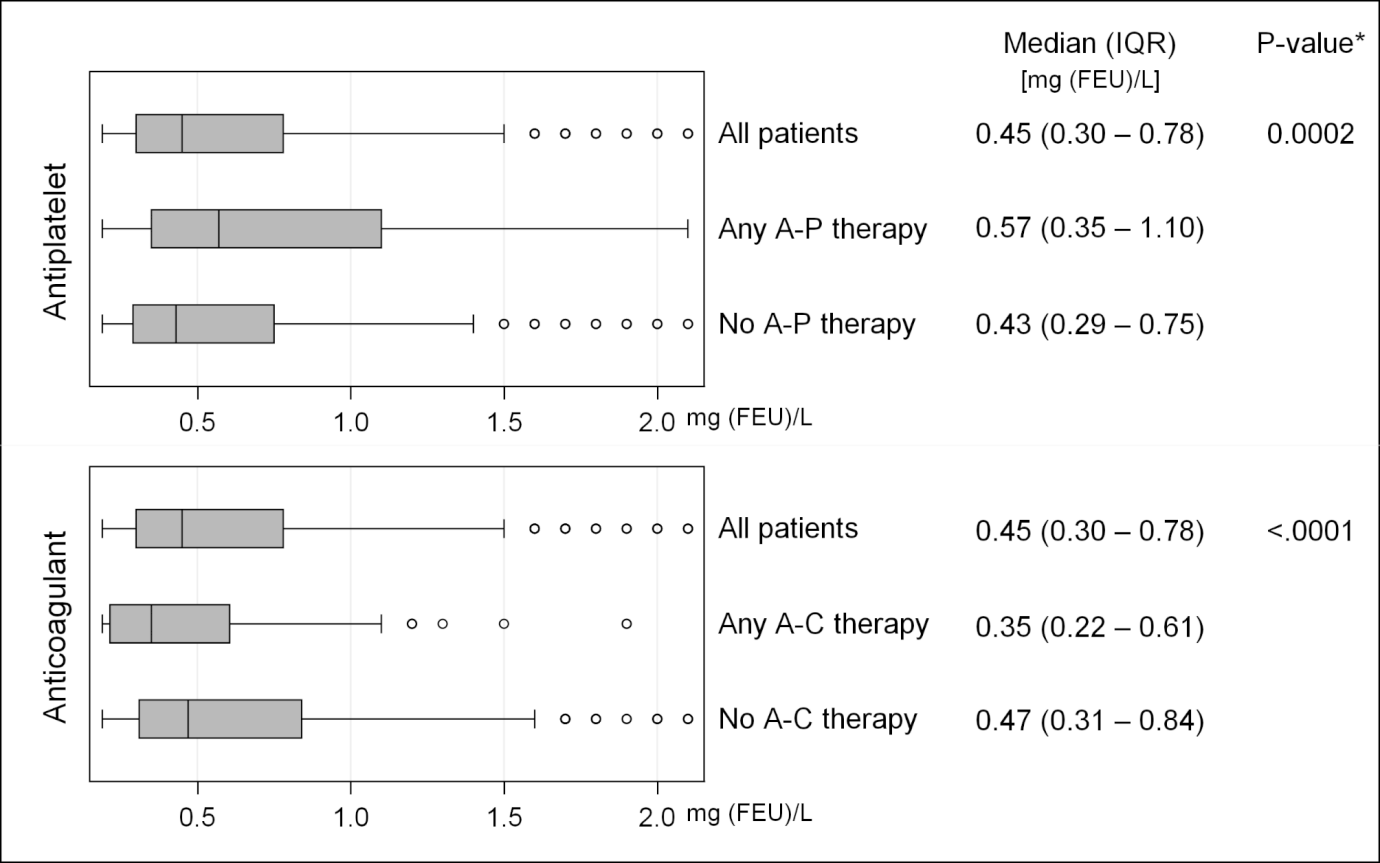
D-dimer levels in users of antiplatelet and anticoagulant treatment. (Box and whiskers plots showing D-dimer levels between users of antiplatelet (A-P) and anticoagulant (A-C) treatment. IQR: Interquartile range, FEU: Fibrin equivalent units. *Wilcoxon Rank-Sum test)

D-dimer levels did not vary significantly between current smokers, former smokers and never smokers (Fig. 3). An explorative Wilcoxon Rank Sum test comparing never smokers to a composite of former and current smokers also showed no difference between the groups (p = 0.067). Users of antiplatelet treatment had higher levels of D-dimer than non-users (median difference 0.14 mg (FEU)/L, p = 0.0002), whereas users of anticoagulant treatment had lower D-dimer levels (median difference 0.12 mg (FEU)/ L, p < 0.0001), see Fig. 4.

D-dimer levels had a weak but significant correlation with age (Spearman Correlation Coefficient: 0.32, p < 0.0001).

### Post-hoc analyses

Mortality rates seemed to increase across the four quartiles of D-dimer (Table 2). The test for trend was p < 0.0001.

**Table 2:** Mortality rates across the four quartiles of D-dimer. FEU: Fibrin equivalent units, N: Number

	First quartile (< 0.30 mg FEU)/L)	Second quartile (0.30–0.44 mg FEU)/L)	Third quartile (0.45–0.77 mg (FEU)/L)	Fourth quartile (≥ 0.78 mg (FEU)/L)
**Follow-up** **(person-years)**	3.87	6.15	8.99	14.72
**Deaths** **(n)**	7	12	17	29
**Mortality rate** **(%)**	4.7	6.4	11.1	18.1

## Discussion

In this study we used the routinely measured D-dimer samples from our COPD out-patients with moderate to severe COPD. We found an association between patients with high D-dimer levels and risk of death; however, this finding was not robust to confounder adjustment. In addition, we found an association between patients with high D-dimer levels and proportion of days alive and out of hospital, where the association seemed more evident throughout confounder adjustment.

Husebø et al. have reported that stable state D-dimer predicted all-cause mortality in their population of COPD patients, especially cardiovascular and pulmonary mortality [13]. These results could seem different to ours, however we did find numerically a larger risk of death among those with high D-dimer and thus chance findings could be an explanation for the differences in p-values. Further, differences in the multivariable models or a difference in the population of COPD patients could contribute. In acute exacerbations of COPD, the results are conflicting, with two studies finding a significant association between D-dimer and long-term mortality, where a third found no association [20–22].

To the best of our knowledge, this is the first study comparing D-dimer levels to smoking status, antiplatelet treatment and anticoagulative treatment in COPD patients; we found that D-dimer levels were significantly higher among patients with concomitant P2Y12-inhibitor treatment. Opposite, D-dimer levels were significantly lower in patients receiving anticoagulant. D-dimer level did not seem to be affected by smoking status in outpatients with COPD. It is possible, that the difference seen in D-dimer levels in patients receiving P2Y12-inhibitor treatment is a consequence of the underlying diseases, and that the difference in patients with anticoagulant treatment is a consequence of the mechanism of action of the drugs.

Our study has several strengths. The availability of laboratory data on registry data ensures a homogenous, unselected study population ensuring a high degree of generalizability. Furthermore, we have a very high degree of follow-up (> 99%) for both the primary outcome of all-cause mortality as well as the secondary outcome of days alive and out of hospital. Additionally, we have a high availability of data on various confounders, with complete data on comorbidities and medication.

It also has limitations. First, premises for pre-study our power calculation were not fulfilled, as we observed a lower risk of death in the total population than expected; thus, the study is possibly underpowered. In addition, our exclusion criteria led to smaller groups possibly contributing to the inconclusiveness of the results. Secondly, we have some missing data; however, these are only for confounding (CRP) or baseline (Body Mass Index, FEV_1_, and smoking status) variables and are sparse.

Both increased mortality and morbidity, including VTE and other cardiovascular diseases, are evident in COPD patients [2–4, 28]. Additionally, increased mortality has been observed in patients with COPD and concomitant VTE [6, 7]. Consequently, it is important to clarify the relationship between COPD and cardiovascular disorders, with the need for mechanistic and prognostic markers to further phenotype this heterogenous patient group. Due to the mechanisms of D-dimer elevation, we find it most plausible that the association exists, however with a lower magnitude than has been found in healthy people. Seeing as D-dimer previously has been linked to all-cause mortality in patients with stable COPD, which is supported in part by our analyses, the pathogenesis of D-dimer elevation is highly relevant in patients with COPD. Contrary, the use of the specific biomarker D-dimer in the clinical setting does not seem relevant taking our results into account, as the magnitude of the possible association seems weak.

In conclusion, we investigated the association between D-dimer and all-cause mortality and days alive and out of hospital. We found no strong signal of excess risk of death from all causes among COPD outpatients with the highest D-dimer. The total mortality in the study population was lower than expected, which is a limitation; however, the signal was not strong, and our data do not support unselected risk stratification of COPD outpatients with D-dimer. It seems unlikely that more events, and thus stronger power, would change this conclusion, even if the p-value decreased in the adjusted analyses.

## Electronic supplementary material

Below is the link to the electronic supplementary material.

Additional File 1: Definition of diagnoses according to International Classification of Disease (ICD-10) codes and definition of treatments according to Anatomical Therapeutic Chemical (ATC)-codes. Description of data: A table containing definition of diagnoses (ICD-codes) and treatments (ATC-codes).

Additional File 2: Full information on the Cox proportional hazards regressions. Description of data: A table containing the full information on the Cox proportional hazards regressions mentioned in the manuscript.

Additional File 3: Full information on the multiple linear regressions for pDAOH. Description of data: A table containing the full information in the multiple linear regression models mentioned in the manuscript.

Additional File 4: Full information on the multiple linear regressions for the participants with 365 days possible follow-up. Description of data: A table containing the full information in the multiple linear regression models for the participants with 365 days possible follow-up, with the unit days.

## Data Availability

The data that support the findings of this study are available from The Danish Healthy Data Authority. Restrictions apply to the availability of these data, which were used under license for this study. Data are available from the The Danish Healthy Data Authority following application and approval from The Danish Healthy Data Authority.

## Abbreviations

ATC: Anatomical Therapeutic Chemical Classification
BMI: Body Mass Index
CI: Confidence Interval
COPD: Chronic Obstructive Pulmonary Disease
CRP: C-reactive protein
CVD: Cardiovascular diseases
pDAOH: Percentage of days alive and out of hospital during follow-up
PP: Percentage point
FEU: Fibrin Equivalent Units
FEV_1_: Forced Expiratory Volume in one second
HR: Hazard Ratio
ICD-10: International Classification of Disease-10
ICS: Inhaled Corticosteroids
IQR: Interquartile Range
VTE: Venous thromboembolism
