# Supplementary material for: Fibrin degradation products and survival in patients with chronic obstructive pulmonary disease: a protocolized prospective observational study
Source: Respir Res. 2023 Jun 27;24:172. doi: 10.1186/s12931-023-02472-9 (PMC10294503; doi:10.1186/s12931-023-02472-9)
Supplement: Supplementary file 1 — Additional File 1: Definition of diagnoses according to International Classification of Disease (ICD-10) codes and definition of treatments according to Anatomical Therapeutic Chemical (ATC)-codes. Description of data: A table containing definition of diagnoses (ICD-codes) and treatments (ATC-codes). [file 12931_2023_2472_MOESM1_ESM.docx]

**Additional File 1:**

Definition of diagnoses according to International Classification of Disease (ICD-10) codes and definition of treatments according to Anatomical Therapeutic Chemical (ATC)-codes.

| **Diagnoses** | **ICD-codes** |
| --- | --- |
| Myocardial infarction | I21.*, I22.*, I25.2 |
| Congestive heart failure | I109.9, I11.0, I13.0, I13.2, I25.5, I42.0, I42.5 – I42.9, I43.*, I50.*, P29.0 |
| Cerebro vascular disease | G45.*, G46.*, H34.0, I60.* – I69.* |
| Dementia | F00.* –F03.* , F05.1, G30.*, G31.1 |
| Rheumatic disease | M05.*, M06.*, M31.5, M32.* – M34.*, M35.1, M35.3, M36.0 |
| Peptic ulcer disease | K25.* – K28.* |
| Liver disease (moderate-severe) | I85.0, I85.9, I86.4, I98.2, K70.4, K71.1, K72.1, K72.9, K76.5, K76.6, K76.7 |
| Diabetes mellitus | E10.* – E14.* |
| Moderate to severe renal disease | I12.0, I13.1, N03.2 – N03.7, N05.2 – N05.7, N18.*, N19.*, N25.0, Z49.0 – Z49.2, Z94.0, Z99.2 |
| Any malignancy | C00.*– C26.*, C30.* – C34.*, C37.* – C41.*, C43.*, C45.* – C58.*, C60.* – C76.*, C77.* – C80.*, C81.* – C85.*, C88.*, C90.* – C97.* |
|  | |
| **Treatments** | **ATC-codes** |
| Anticoagulant | B01AA03, B01AA04, B01AE07, B01AF01, B01AF02, B01AF03 |
| Acetylsalicylic acid | B01AC06 |
| P2Y12-inhibitors | B01AC04, B01AC07, B01AC22, B01AC24 |
| Prednisolone | H02AB06, H02AB09 |
| Inhaled corticosteroid treatment | R03AK, R03AL08, R03AL09, R03AL11, R03AL12, R03BA. |
| Inhaled long-acting muscarinic antagonist | R03AL, R03BB |
| Inhaled long-acting β2-agonist | R03AL, R03AC1, R03AK |
