# Supplementary material for: Fibrin degradation products and survival in patients with chronic obstructive pulmonary disease: a protocolized prospective observational study
Source: Respir Res. 2023 Jun 27;24:172. doi: 10.1186/s12931-023-02472-9 (PMC10294503; doi:10.1186/s12931-023-02472-9)
Supplement: Supplementary file 2 — Additional File 2: Full information on the Cox proportional hazards regressions. Description of data: A table containing the full information on the Cox proportional hazards regressions mentioned in the manuscript. [file 12931_2023_2472_MOESM2_ESM.docx]

**Additional File 2**

Full information on the Cox proportional hazards regressions

|  |  | **95% Confidence interval** | |  |
| --- | --- | --- | --- | --- |
| **Unadjusted** | **HR** | **Lower** | **Upper** | **P-value** |
| D-dimer (high) | 2,272 | 1,103 | 4,677 | 0,026 |
|  |  |  |  |  |
|  |  | **95% Confidence interval** | |  |
| **Age and sex adjusted** | **HR** | **Lower** | **Upper** | **P-value** |
| D-dimer (high) | 1,999 | 0,956 | 4,181 | 0,066 |
| Age | 1,032 | 0,992 | 1,074 | 0,113 |
| Sex (male) | 1,690 | 0,818 | 3,491 | 0,157 |
|  |  |  |  |  |
|  |  | **95% Confidence interval** | |  |
| **Fully adjusted** | **HR** | **Lower** | **Upper** | **P-value** |
| D-dimer (high) | 1,809 | 0,838 | 3,902 | 0,131 |
| Age | 1,033 | 0,993 | 1,074 | 0,109 |
| Sex (male) | 1,671 | 0,802 | 3,483 | 0,170 |
| CRP | 1,010 | 0,988 | 1,032 | 0,392 |
| Previous high dose prednisolone | 0,725 | 0,249 | 2,112 | 0,556 |
| Previous low dose prednisolone | 0,830 | 0,111 | 6,235 | 0,857 |
| Previous ICS use | 1,559 | 0,728 | 3,339 | 0,253 |
|  |  |  |  |  |
|  |  | **95% Confidence interval** | |  |
| **Anticoagulant interaction** | **HR** | **Lower** | **Upper** | **P-value** |
| D-dimer (high) | 2,153 | 0,923 | 5,024 | 0,076 |
| Age | 1,031 | 0,989 | 1,075 | 0,148 |
| Sex (male) | 1,673 | 0,794 | 3,525 | 0,176 |
| CRP | 1,009 | 0,987 | 1,032 | 0,405 |
| Previous high dose prednisolone | 0,733 | 0,252 | 2,132 | 0,568 |
| Previous low dose prednisolone | 0,828 | 0,110 | 6,224 | 0,854 |
| Previous ICS use | 1,628 | 0,757 | 3,501 | 0,213 |
| Anticoagulant treatment | 1,528 | 0,515 | 4,534 | 0,445 |
| D-dimer (high) / Anticoagulant treatment interaction | 0,356 | 0,036 | 3,568 | 0,380 |
|  |  |  |  |  |
|  |  | **95% Confidence interval** | |  |
| **P2Y12-inhibitor interaction** | **HR** | **Lower** | **Upper** | **P-value** |
| D-dimer (high) | 1,971 | 0,858 | 4,530 | 0,110 |
| Age | 1,030 | 0,990 | 1,071 | 0,150 |
| Sex (male) | 1,640 | 0,785 | 3,426 | 0,188 |
| CRP | 1,008 | 0,985 | 1,032 | 0,493 |
| Previous high dose prednisolone | 0,693 | 0,237 | 2,026 | 0,502 |
| Previous low dose prednisolone | 0,859 | 0,114 | 6,468 | 0,883 |
| Previous ICS use | 1,576 | 0,735 | 3,379 | 0,243 |
| P2Y12-inhibitor treatment | 3,610 | 1,026 | 12,701 | 0,045 |
| D-dimer (high) / P2Y12-inhibitor interaction | 0,519 | 0,072 | 3,718 | 0,513 |
