# Supplementary material for: Fibrin degradation products and survival in patients with chronic obstructive pulmonary disease: a protocolized prospective observational study
Source: Respir Res. 2023 Jun 27;24:172. doi: 10.1186/s12931-023-02472-9 (PMC10294503; doi:10.1186/s12931-023-02472-9)
Supplement: Supplementary file 3 — Additional File 3: Full information on the multiple linear regressions for pDAOH. Description of data: A table containing the full information in the multiple linear regression models mentioned in the manuscript. [file 12931_2023_2472_MOESM3_ESM.docx]

**Additional File 3**

Full information on the multiple linear regressions for percentage of follow-up time being alive and out of hospital.

|  |  |  | **95% Confidence interval** | |  |
| --- | --- | --- | --- | --- | --- |
| **Unadjusted** | **Estimate** | **S.E.** | **Lower** | **Upper** | **P-value** |
| Intercept | 98,447 | 0,312 | 97,835 | 99,060 | <0,001 |
| D-dimer | -2,722 | 0,614 | -3,926 | -1,517 | <0,001 |
|  |  |  |  |  |  |
|  |  |  | **95% Confidence interval** | |  |
| **Age and sex adjusted** | **Estimate** | **S.E.** | **Lower** | **Upper** | **P-value** |
| Intercept | 102,221 | 2,179 | 97,949 | 106,492 | <0,001 |
| D-dimer (high) | -2,502 | 0,630 | -3,737 | -1,266 | <0,001 |
| Age | -0,049 | 0,030 | -0,107 | 0,010 | 0,105 |
| Sex (male) | -0,751 | 0,544 | -1,817 | 0,315 | 0,167 |
|  |  |  |  |  |  |
|  |  |  | **95% Confidence interval** | |  |
| **Fully adjusted** | **Estimate** | **S.E.** | **Lower** | **Upper** | **P-value** |
| Intercept | 102,883 | 2,159 | 98,651 | 107,114 | <0,001 |
| D-dimer (high) | -1,652 | 0,636 | -2,899 | -0,405 | 0,009 |
| Age | -0,044 | 0,029 | -0,102 | 0,014 | 0,138 |
| Sex (male) | -0,839 | 0,535 | -1,887 | 0,208 | 0,116 |
| CRP | -0,065 | 0,012 | -0,089 | -0,042 | <0,001 |
| Previous high dose prednisolone | 0,036 | 0,712 | -1,360 | 1,432 | 0,96 |
| Previous low dose prednisolone | -1,675 | 1,467 | -4,550 | 1,201 | 0,254 |
| Previous ICS use | -0,788 | 0,538 | -1,842 | 0,267 | 0,143 |
|  |  |  |  |  |  |
|  |  |  | **95% Confidence interval** | |  |
| **Anticoagulant interaction** | **Estimate** | **S.E.** | **Lower** | **Upper** | **P-value** |
| Intercept | 102,104 | 2,197 | 97,798 | 106,410 | <0,001 |
| D-dimer (high) | -1,519 | 0,683 | -2,859 | -0,180 | 0,026 |
| Age | -0,031 | 0,031 | -0,091 | 0,029 | 0,311 |
| Sex (male) | -0,763 | 0,535 | -1,812 | 0,285 | 0,154 |
| CRP | -0,061 | 0,012 | -0,085 | -0,038 | <0,001 |
| Previous high dose prednisolone | 0,003 | 0,711 | -1,390 | 1,396 | 0,997 |
| Previous low dose prednisolone | -1,621 | 1,464 | -4,490 | 1,247 | 0,268 |
| Previous ICS use | -0,826 | 0,540 | -1,884 | 0,233 | 0,126 |
| Anticoagulant treatment | -0,896 | 0,820 | -2,504 | 0,711 | 0,274 |
| D-dimer (high) / Anticoagulant treatment interaction | -2,626 | 1,805 | -6,163 | 0,912 | 0,146 |
|  |  |  |  |  |  |
|  |  |  |  |  |  |
|  |  |  | **95% Confidence interval** | |  |
| **P2Y12-inhibitor interaction** | **Estimate** | **S.E.** | **Lower** | **Upper** | **P-value** |
| Intercept | 102,909 | 2,164 | 98,667 | 107,151 | <0,001 |
| D-dimer (high) | -1,690 | 0,662 | -2,987 | -0,392 | 0,011 |
| Age | -0,044 | 0,030 | -0,102 | 0,014 | 0,136 |
| Sex (male) | -0,835 | 0,536 | -1,885 | 0,215 | 0,119 |
| CRP | -0,065 | 0,012 | -0,089 | -0,042 | <0,001 |
| Previous high dose prednisolone | 0,038 | 0,714 | -1,362 | 1,438 | 0,957 |
| Previous low dose prednisolone | -1,656 | 1,471 | -4,540 | 1,227 | 0,26 |
| Previous ICS use | -0,780 | 0,540 | -1,837 | 0,278 | 0,149 |
| P2Y12-inhibitor treatment | -0,061 | 1,291 | -2,592 | 2,470 | 0,962 |
| D-dimer (high) / P2Y12-inhibitor interaction | 0,471 | 2,264 | -3,966 | 4,908 | 0,835 |
