# Supplementary material for: Fibrin degradation products and survival in patients with chronic obstructive pulmonary disease: a protocolized prospective observational study
Source: Respir Res. 2023 Jun 27;24:172. doi: 10.1186/s12931-023-02472-9 (PMC10294503; doi:10.1186/s12931-023-02472-9)
Supplement: Supplementary file 4 — Additional File 4: Full information on the multiple linear regressions for the participants with 365 days possible follow-up. Description of data: A table containing the full information in the multiple linear regression models for the participants with 365 days possible follow-up, with the unit days. [file 12931_2023_2472_MOESM4_ESM.docx]

**Additional File 4**

Full information on the multiple linear regressions for the participants with 365 days possible follow-up time (n=461). Outcome: Days alive and out of hospital.

|  |  |  | **95% Confidence interval** | |  |
| --- | --- | --- | --- | --- | --- |
| **Unadjusted** | **Estimate** | **S.E.** | **Lower** | **Upper** | **P-value** |
| Intercept | 357,833 | 2,162 | 353,595 | 362,071 | <0,001 |
| D-dimer | -9,146 | 4,365 | -17,701 | -0,591 | 0,036 |
|  |  |  |  |  |  |
|  |  |  | **95% Confidence interval** |  |  |
| **Age and sex adjusted** | **Estimate** | **S.E.** | **Lower** | **Upper** | **P-value** |
| (Intercept) | 375,270 | 14,555 | 346,742 | 403,798 | <0,001 |
| D-dimer (high) | -8,195 | 4,438 | -16,894 | 0,504 | 0,065 |
| Age | -0,241 | 0,200 | -0,634 | 0,152 | 0,229 |
| Sex (male) | -1,295 | 3,818 | -8,777 | 6,188 | 0,734 |
|  |  |  |  |  |  |
|  |  |  | **95% Confidence interval** |  |  |
| **Fully adjusted** | **Estimate** | **S.E.** | **Lower** | **Upper** | **P-value** |
| (Intercept) | 384,086 | 14,666 | 355,341 | 412,832 | <0,001 |
| D-dimer (high) | -4,414 | 4,661 | -13,550 | 4,721 | 0,344 |
| Age | -0,275 | 0,199 | -0,665 | 0,115 | 0,167 |
| Sex (male) | -1,373 | 3,819 | -8,858 | 6,112 | 0,719 |
| CRP | -0,346 | 0,162 | -0,662 | -0,029 | 0,033 |
| Previous high dose prednisolone | -0,864 | 5,547 | -11,735 | 10,007 | 0,876 |
| Previous low dose prednisolone | 15,050 | 13,154 | -10,731 | 40,832 | 0,253 |
| Previous ICS use | -9,492 | 3,861 | -17,061 | -1,924 | 0,014 |
|  |  |  |  |  |  |
|  |  |  | **95% Confidence interval** |  |  |
| **Anticoagulant interaction** | **Estimate** | **S.E.** | **Lower** | **Upper** | **P-value** |
| (Intercept) | 378,039 | 15,059 | 348,523 | 407,555 | <0,001 |
| D-dimer (high) | -4,564 | 5,017 | -14,397 | 5,269 | 0,363 |
| Age | -0,173 | 0,208 | -0,581 | 0,235 | 0,407 |
| Sex (male) | -0,462 | 3,854 | -8,016 | 7,092 | 0,905 |
| CRP | -0,340 | 0,161 | -0,656 | -0,023 | 0,035 |
| Previous high dose prednisolone | -0,438 | 5,544 | -11,304 | 10,428 | 0,937 |
| Previous low dose prednisolone | 13,049 | 13,190 | -12,803 | 38,900 | 0,323 |
| Previous ICS use | -9,671 | 3,868 | -17,253 | -2,089 | 0,012 |
| Anticoagulant treatment | -7,603 | 5,630 | -18,637 | 3,431 | 0,177 |
| D-dimer (high) / Anticoagulant treatment interaction | -5,117 | 12,184 | -28,998 | 18,764 | 0,674 |
|  |  |  |  |  |  |
|  |  |  | **95% Confidence interval** |  |  |
| **P2Y12-inhibitor interaction** | **Estimate** | **S.E.** | **Lower** | **Upper** | **P-value** |
| (Intercept) | 383,624 | 14,674 | 354,864 | 412,384 | <0,001 |
| D-dimer (high) | -5,524 | 4,788 | -14,909 | 3,861 | 0,249 |
| Age | -0,259 | 0,199 | -0,650 | 0,132 | 0,194 |
| Sex (male) | -1,178 | 3,807 | -8,639 | 6,283 | 0,757 |
| CRP | -0,341 | 0,161 | -0,657 | -0,025 | 0,034 |
| Previous high dose prednisolone | 0,123 | 5,544 | -10,743 | 10,988 | 0,982 |
| Previous low dose prednisolone | 14,685 | 13,124 | -11,038 | 40,408 | 0,263 |
| Previous ICS use | -9,397 | 3,847 | -16,937 | -1,856 | 0,015 |
| P2Y12-inhibitor treatment | -23,148 | 10,850 | -44,414 | -1,882 | 0,033 |
| D-dimer (high) / P2Y12-inhibitor interaction | 23,929 | 17,144 | -9,674 | 57,531 | 0,163 |
